# Enhanced Th2 immune response in milk-allergic children against the major milk proteins as the starting point for an OIT protocol

**DOI:** 10.1186/2045-7022-3-S3-P51

**Published:** 2013-07-25

**Authors:** M Reche, T Valbuena, C Pascual, A Padial, L Perezábad, R López-Fandiño, E Molina, I López Expósito

**Affiliations:** 1 Allergy Service, Infanta Sofia Hospital, Madrid, Spain; 2 Bioactivity and Food Analysis, Institute for Food Science Research (CIAL) (CSIC-UAM), Madrid, Spain

## Background

Milk allergy is the most common food allergy in infants and young children. Typical milk allergy symptoms involve hives, vomiting, or even anaphylaxis. For cow’s milk, strict avoidance is difficult because milk proteins are very ubiquitous. Oral immunotherapy (OIT) is an effective therapeutic option to induce milk oral tolerance. However, the immune mechanisms underlying this treatment have not been fully identified yet. The aim of this study was to compare the clinical and immune responses against several milk allergens of a pediatric population before an OIT protocol with a non-atopic population of the same age range.

## Methods

A group of 9 patients with IgE-mediated milk allergy, confirmed by positive double blind placebo controlled test (DBPCT), was compared with a group of 8 non-atopic people on a milk-containing diet. The mean age of milk-allergic patients was 4.7 years compared with 6.0 years in the control group. No significant differences regarding sex or age were found between them. In both groups, PBMCs were isolated from peripheral blood and stimulated during 7 days with β-casein (β-Cn), α-lactalbumin (α-La) and β-lactoglobulin (β-Lg). Culture supernatants were analyzed for IL-5, IL-13, IL-1β, IFN-γ, TNF-α and IL-10 production by Cytometric Bead Array (CBA).

## Results

During the DBPCT with milk, 6 milk-allergic patients had urticaria, 1 referred digestive symptoms and 1 respiratory symptoms. The mean positive dose in the DBPCT was 15.4 mL of milk, within a range of 0.3-64 mL. Specific IgE measurements were, on average, 37.9 kU/L for milk, 40.1 kU/L for casein, 28.63 kU/L for α-La and 17.45 kU/L for β-Lg.

CBA analysis revealed a significantly increased production of β-Lg and β-Cn-specific IL-5 and IL-13 (P<0.05), together with a trend towards lower α-La (P=0.09) and β-Lg (P=0.06)-stimulated TNF-α, in milk-allergic patients as compared with control children.

## Conclusion

Under the conditions studied, milk-allergic patients showed a significantly enhanced Th2 cytokine production after stimulation with milk allergens in comparison with non-atopic controls. In addition, a trend towards lower TNF-α production was observed. From the results obtained, it can be concluded that stimulation with both β-Lg and β-Cas shifted the T-cells phenotype to a Th2 profile, which is well correlated with the clinical response observed during the DBPCT. Studies are underway aiming to establish the role of the evolution of the cytokine profile during an OIT protocol.

## Disclosure of interest

None declared.

